# Individual differences in goal adjustment: convergence and divergence among three theoretical models

**DOI:** 10.3389/fpsyg.2024.1288667

**Published:** 2024-05-30

**Authors:** Cathleen Kappes, Werner Greve

**Affiliations:** University of Hildesheim, Hildesheim, Germany

**Keywords:** goal disengagement, accommodation, secondary control processes, goal adjustment, well-being, emotion regulation

## Abstract

**Introduction:**

Individual differences in dealing with unattainable goals or resource-consumptive goal pursuit are conceptualized as goal adjustment processes in three theoretical approaches: accommodative coping (*two-process model of developmental regulation*), compensatory secondary coping (*motivational theory of life-span development*), and goal disengagement and goal reengagement (*goal adjustment theory*). The aim of this paper is to conceptually and empirically analyze convergences and divergences between the three approaches as well as their relationship with indicators of well-being and their intersections with cognitive emotion regulation.

**Methods:**

The empirical study is based on a cross-sectional online survey (*N* = 433; *M* = 28.9 years, *SD* = 8.4 years; 50% female).

**Results:**

The conceptual analysis yields clear convergences, but also differences. Empirically, clear but partially non-redundant relationships between the concepts are found in structural equation models. Accommodative coping showed the strongest correlations with measures of well-being and cognitive emotion regulation. When all goal adjustment measures are included simultaneously as predictors of well-being, accommodation remains as the strongest predictor (and partly goal reengagement as well), while goal disengagement shows opposing relationships with most measures of well-being.

**Discussion:**

We discuss the lessons learnt from these findings and conclude by proposing future avenues to examine goal adjustment processes.

## Introduction

Pursuing personally important goals and plans gives meaning and structure to life and maintains its quality (Brandtstädter, 2007; Mens et al., 2015). In the course of life, however, everyone is confronted with goals that are difficult or not (any longer) attainable. Increasing losses with age, e.g., of social or physical resources, may limit the variety or number of goals that can be achieved (Brandtstädter and Rothermund, 2002; Mens et al., 2015). Moreover, biological or sociocultural deadlines as well as personal time limits may also restrict the ability to pursue important life goals in younger years (Heckhausen et al., 2010). Since such goal blockages threaten the individuals’ quality of life, successfully overcoming goal blockages is an increasingly important resource for successful development across the lifespan.

In recent decades, a growing body of research has provided evidence that, besides strategies of persistent goal pursuit, adaptive self-regulatory processes of goal adjustment may prove functional with respect to various facets of quality of life (e.g., Brandtstädter and Rothermund, 2002; Wrosch et al., 2003a,b; Heckhausen et al., 2010). These processes can support individuals in their coping with goal blockages, critical life events and transitions, and mitigate their negative psychological and physical consequences (e.g., Brandtstädter, 2007; Heckhausen et al., 2019; meta-analysis by Barlow et al., 2019). Two particularly influential models that address both goal pursuit and goal adjustment across the lifespan are the *two-process model of developmental regulation* (Brandtstädter and Renner, 1990; Brandtstädter and Rothermund, 2002; Brandtstädter, 2007) and the *motivational theory of life-span development* and its theoretical precursors (Heckhausen and Schulz, 1993, 1995; Schulz and Heckhausen, 1996; Heckhausen et al., 2010). Additionally, the *goal adjustment theory* of Wrosch et al. (2003a,b) particularly focusses on goal disengagement and goal reengagement as processes relevant for coping with goal blockages. All three theories examine basically the same functional processes: Coping with the experience that a goal has become (subjectively) unattainable or too resource-consuming. However, there are only a few approaches to clarify the interrelationship between the *two-process model* and *the motivational theory of life-span development* conceptually [Boerner and Jopp, 2007; and commentaries to their analysis by Poulin et al. (2005), Greve and Wentura (2007), and Riediger and Ebner (2007)], while the intersections with the *goal adjustment theory* have not been systematically considered yet.

Empirically, Haase et al. (2013) compared different goal regulation theories (for another empirical comparison, see Wahl et al., 2005). In two cross-sectional studies^1^ comparing the two-process model of developmental regulation and the motivational theory of life-span development, they demonstrated that parceled items of the (sub)scales assessing processes of accommodative coping (Brandtstädter and Renner, 1990; Brandtstädter and Rothermund, 2002) and compensatory secondary control processes (Heckhausen et al., 2010), respectively, loaded on one factor termed *goal disengagement* in confirmatory factor analyses (Wahl et al., 2005, also obtained a strong positive correlation between both scales). Assessment of processes of the goal adjustment theory was not included in their study.

Moreover, although a conceptual resemblance of goal adjustment processes with cognitive emotion regulatory processes, such as positive reappraisal (*cf*. Thomsen, 2016; Loidl and Leipold, 2019), seems plausible, this relationship was rarely empirically tested.

Accordingly, the aims of this paper are to (1) contribute to a conceptual clarification of the relationship between different theoretical approaches to goal adjustment processes and cognitive emotion regulation, (2) provide empirical data for an investigation of intersections and divergences of the respective assessment instruments and a potential integration of these approaches, and (3) point out future avenues for this field of research. To achieve these aims, we will first introduce each theory’s basic tenets with a focus on processes of goal adjustment [although the two-process model of developmental regulation (Brandtstädter and Rothermund, 2002) and the motivational theory of life-span development (Heckhausen et al., 2010) consider processes of tenacious goal pursuit as well]. Ensuing, we outline commonalities and differences concerning each theory’s conceptualization of processes, their functionality, and their relationship with cognitive emotion regulation.

### Basic tenets of goal adjustment theories

According to the two-process model or dual model of assimilative and accommodative coping (Brandtstädter and Renner, 1990; Brandtstädter and Rothermund, 2002; Brandtstädter, 2007), an essential principle underlying developmental regulation is the maintenance of the individual’s self as stable, consistent, and positive as possible over the lifespan (Brandtstädter and Greve, 1994). Central to the model is the argument that problem situations, such as goal blockages, developmental losses or other aversive events, represent discrepancies between a present subjectively perceived state of the situation or self (actual state) and a desired state (target state) (Brandtstädter, 1989, 2011; Brandtstädter and Rothermund, 2002). If these actual-target discrepancies cannot be reduced, emotional stress may result (Brandtstädter, 2011). According to Brandtstädter and Renner (1990) and Brandtstädter (2011), such discrepancies and possibly associated negative emotions can be reduced or even resolved in two different ways: On the one hand, individuals try to alter (improve) the actual state toward the target state (assimilative coping mode). On the other hand, the discrepancy can be reduced by adapting the desired state to the actual state (accommodative coping mode). It is the latter that we want to focus on for the purpose of this paper.

Another influential approach to developmental regulation across the lifespan is the motivational theory of life-span development (Heckhausen et al., 2010), which is an extension of previous theoretical approaches (life-span theory of control, Heckhausen and Schulz, 1995; model of optimization by primary and secondary control; Heckhausen and Schulz, 1993; Schulz and Heckhausen, 1996; Heckhausen, 1997; action-phase model of developmental regulation, Heckhausen, 1999). The motivational theory of life-span development distinguishes between primary and secondary control processes. Central to the theory is the assumption that individuals strive to maximize control over their environment throughout life (Heckhausen and Schulz, 1995, 1999). This so-called primary control refers to actively influencing external factors to match the individual’s aspired goals, needs, and desires. In contrast, secondary control describes internal cognitive processes that produce changes within the individual in order to achieve an adaptation to the given external conditions (Heckhausen and Schulz, 1995). Although the term control suggests a conscious process, secondary control processes are generally thought to occur unconsciously (Poulin et al., 2005). The function of secondary adjustment processes is to minimize losses as well as to maintain or regain primary control (Heckhausen and Schulz, 1995). It is argued that the functional primacy of primary control is evolutionary in that it helped to provide selection advantages in terms of reproduction and survival (Heckhausen and Schulz, 1999; Poulin et al., 2005). Accordingly, it is argued that selection and compensation can be functional or dysfunctional depending on whether the chosen strategy is conducive to primary control or not (Heckhausen and Schulz, 1993). In this model, compensatory secondary control processes are the response to unattainable goals.

Third, the goal adjustment theory solely deals with the aspect of turning away from unattainable goals (Wrosch et al., 2003a,b; Mens et al., 2015). This theory assumes that an exclusive focus on goal pursuit may have negative effects on psychological well-being, as investment in unattainable goals may be associated with stress and negative cognitions and feelings about the unattained goal due to repeated failure, among other factors (Mens et al., 2015). According to goal adjustment theory, an adaptive response to an unattainable goal is therefore goal adjustment consisting of disengagement from the unattainable goal and reengagement in alternative attainable goals (Wrosch et al., 2003a; Mens et al., 2015).

### Processes of goal adjustment and their measurement

While all three approaches propose regulatory processes in coping with unattainable goals or dwindling resources for goal pursuit, respectively, they differ in their emphasis on relevant processes, how goal disengagement itself is conceptualized, and how individual differences are measured. Table 1 gives an overview of the proposed processes.

**Table 1:** Comparison of the regulatory processes of the theoretical approaches.

Theoretical approach		
Two-process model: accommodation	Motivational theory of life-span development: compensatory secondary control	Goal adjustment theory: goal disengagement and goal reengagement
Disengagement from the unattainable goal Devaluing an unattainable goal Changing one’s aspirations Positively reinterpreting the negative event Redirecting resources to other attainable goals	Distancing from goal: devalue chosen goal, downgrade importance of goal, enhance value of conflicting goals Self-protection: self-serving comparisons (down-ward social comparisons, temporal or dimensional comparison), self-esteem-protective attributions of failure	*Goal disengagement* Cessation of behavioral efforts to achieve the goal Dissolution of goal commitment *Goal reengagement* Identification of alternative attainable goals Commitment to new goals Effort to achieve these new goals

Two-process model of developmental regulation (Brandtstädter and Rothermund, 2002; Brandtstädter, 2007). Motivational theory of life-span development (Heckhausen et al., 2010, 2019). Goal adjustment theory (Wrosch et al., 2003a,b; Wrosch and Scheier, 2020).

Accommodative coping as a category or mode of coping (Greve and Kappes, 2023) refers to a number of processes by which personal preferences and goals are adjusted to the given possibilities or constraints for action (Brandtstädter and Renner, 1990; Brandtstädter, 2011). This is possible, for example, by devaluing an unattainable goal, changing one’s aspirations, positively reinterpreting the negative event, disengagement from the unattainable goal, or redirecting resources to attainable goals (Brandtstädter, 2007). Because the accommodative coping mode focuses less on active problem solving and more on changing the structure of cognitions and evaluations in order to experience the current situation less negatively, the effect of accommodative processes is a kind of dissolution of the threatening constellation. According to the model, accommodative processes need not be conscious or volitional (Brandtstädter and Renner, 1990; Brandtstädter and Rothermund, 2002).

On the basis of the Rubicon model (Heckhausen and Gollwitzer, 1987), Heckhausen (1999) conceptualized the action phase model of developmental regulation. Here, a cyclical structure of action phases is postulated, which includes goal selection, goal commitment, goal disengagement, and subsequent recommitment to adapted or new goals. Specific control strategies of selection and compensation are assigned to each of these action phases. If the goal is not attainable even by compensatory primary control (i.e., the use of external means such as the assistance of others, the use of various tools, or the use of unusual ways to achieve the goal), strategies of compensatory secondary control can be used to protect against the negative effects of failure. These strategies are the focus of the present article. They include internal adaptations, such as abandoning the desired goal, strategic social comparisons, or self-esteem-protective attributions of cause (Heckhausen and Schulz, 1993; Heckhausen et al., 2019).

In the goal adjustment theory, goal disengagement and goal reengagement are differentiated (Wrosch et al., 2003a,b). Goal disengagement distinguishes two components: the abandonment of efforts to achieve a blocked goal and the abandonment of commitment to or engagement for that goal (Mens et al., 2015). Accordingly, it is essential that goal disengagement does not consist exclusively of the cessation of behavioral efforts to achieve the goal, but that complete disengagement also requires the dissolution of goal commitment (Wrosch et al., 2003a). Wrosch et al. (2003a) proposed that various factors can facilitate goal disengagement, such as the use of self-protective processes (e.g., downward comparisons), a tendency toward high self-monitoring, or the certainty that a goal is unattainable. In turn, goal disengagement may be hampered by a lack of availability of alternative goals or a tendency to make positively biased judgments (Wrosch et al., 2003a). Importantly, goal disengagement is conceptually separated from processes facilitating it. Goal reengagement comprises three components: First, alternative attainable goals must be identified that also appear personally relevant (Mens et al., 2015). The second component involves commitment to these goals. Third, efforts are made to achieve these new goals.

#### Conceptualization of goal disengagement

Despite the theories’ similarity and convergence with respect to the general idea of dissolution of tensions created by goal blocking through goal adjustment, there are notable differences. First of all, it is important to note that both the two-process model (Brandtstädter and Renner, 1992 Brandtstädter and Rothermund, 2002) and the control theory (Heckhausen et al., 2010, 2019) combine different processes for goal adjustment into one category (accommodation and secondary compensatory control, respectively). Alas, the conceptual level or status of these processes is not always entirely clear. For instance, in the two-process model, goal disengagement is oftentimes referred to as one process (at the same conceptual level) among other processes reducing the actual-target discrepancy (e.g., downward comparison); however, at times it is also conceptualized as the result of these processes (e.g., positively reinterpreting the negative event signals the process of goal disengagement and results in the dissolution of commitment; Brandtstädter and Rothermund, 2002). In contrast, goal adjustment theory (Wrosch et al., 2003a) focuses separately on two particular aspects of goal adjustment (disengagement and reengagement). The emphasis on intentionality and control of specific (or multiple) aspects of goal adjustment (especially goal disengagement) is somewhat more pronounced in control theory, although, as mentioned above, it does not assume full control by the individual (goal adjustment theory is neutral on this point). Neither the control theory nor the two-process model claim to present a complete compilation of the causally relevant or constitutive sub-processes and single processes for goal adjustment (accommodation). Rather, prototypical (sub-)processes are named. It is precisely on this point that the operationalization presented in each case is particularly revealing.

In the two-process-model, individual differences in accommodative coping are measured with the scale *flexible goal adjustment* (FGA; assimilative coping scale: Tenacious Goal Pursuit, TGP; Brandtstädter and Renner, 1990). This scale does not even encompass all facets of the accommodative mode conceptualized in this model (particularly not goal disengagement) and it also does not distinguish between different facets of the accommodative mode. In contrast, Loidl and Leipold (2019) have proposed to differentiate between the five different facets of accommodation. Using their newly developed questionnaire, they found that, while positive reappraisal/personal growth, lowering of aspirations/acceptance, downward comparison, and reorientation are moderately to strongly correlated with each other, disengagement from the goal showed only correlations with lowering of aspirations and reorientation. Moreover, while the other facets were significantly positively associated with measures of well-being and negatively with measures of ill-being, goal disengagement mostly showed no such correlations or even reversed associations. Accordingly, goal disengagement seems to occupy a special position. This conclusion is supported by a recent longitudinal study (Loidl and Leipold, 2022).

Heckhausen et al. (2010, 2019) conceptualize compensatory secondary control processes as entailing “active disengagement in terms of withdrawal of effort and breaking of commitment” (2010, p. 41), supplemented by processes to protect the self from potentially damaging effects of an experience of failure. To capture individual differences in each of the strategies of the whole developmental regulation model, Heckhausen et al. (1998) developed the Optimization in Primary and Secondary Control Scales (OPS-Scales), which measure the five goal regulation strategies by means of subscales. The subscale *compensatory secondary control* (CSC) considers mainly the protective processes proposed concerning these control strategies but also includes items on the ease of goal disengagement.

The latter is one of the two aspects focused in the goal adjustment theory (Wrosch et al., 2003b). Although other processes are assumed as facilitating the process of goal disengagement, the ease of cognitive-affective dissolution of commitment as well as letting go behaviorally is the focus of consideration in Wrosch et al.’s (2003b) theoretical approach. This is also mirrored in the subscale *goal disengagement* (GD; with the two components of abandonment of effort and commitment to an unattainable goal) of the Goal Adjustment Scale (GAS; Wrosch et al., 2003b), which was developed to assess individual differences in goal adjustment capacity.

#### Conceptualization of goal reorientation

Another difference between the theoretical approaches and their operationalization concerns the role assigned to engagement with alternative goals. In the dual-process model, reorientation toward attainable alternative goals or their revaluation is conceptualized as part of the accommodative processes and hence is included in the FGA-scale. Expanding the attentional field toward other goals is deemed a kind of expression of goal disengagement taking place (Brandtstädter and Rothermund, 2002), and it can also be conducive to support further goal disengagement. In Heckhausen et al.’s (2010, 2019) conceptualization, goal reengagement facilitates goal disengagement as well. As such, it is also directly included in the subscale of *compensatory secondary control*. In contrast, Wrosch et al. (2003b) conceptualize goal reengagement as an independent process, which is necessary to find (new) meaning in life. The differentiation between disengagement vs. reengagement is reflected in a separate subscale for *goal reengagement* (GR) of the Goal Adjustment Scale (Wrosch et al., 2003b). This subscale is composed of items on identification, commitment, and pursuit of new goals.

### Goal adjustment and regulatory outcomes

Because the pursuit of blocked or unattainable goals can be associated with negative psychological and physical consequences, it is assumed that adaptive responses to these goal blockages are important or even necessary for the maintenance of well-being (and for successful development). Regulatory mechanisms are claimed to serve this proximal function in all three theories; yet, the theories differ in their basic assumptions about the distal function of adaptive processes. According to Brandtstädter (2001), an essential principle underlying developmental regulation is the maintenance of continuity and consistency of the self across the lifespan. In contrast, according to Heckhausen and Schulz (1995), the essential motivation of the individual is to maximize control over one’s environment (primary control) across the lifespan. Wrosch’s approach (Wrosch and Scheier, 2020) distinguishes different main functions of goal disengagement and goal reorientation: Goal disengagement ability serves to reduce experiences of failure and enables the use of resources for other activities. Goal reengagement creates meaning in life and reduces the thoughts and emotions associated with failure.

Despite these partly diverging assumptions, all three approaches have been demonstrated to be associated with similar outcome measures of well-being (or its absence). Accommodative adaptation processes have been shown to stabilize the sense of self-efficacy, sense of personal control, well-being, self-esteem and life satisfaction as well as to counteract depressive tendencies (Brandtstädter and Rothermund, 2002; Heyl et al., 2007; Rühs et al., 2017; Greve et al., 2018; Marek et al., 2022). Similarly, compensatory secondary control processes have been demonstrated to be negatively and positively associated with well-being depending on contextual variables such as the availability of resources (Heckhausen et al., 2010; Haase et al., 2012; Tomasik and Salmela-Aro, 2012; Grümer et al., 2013). Finally, empirical findings support the assumption that high goal disengagement contributes to a decrease in negative aspects of well-being (e.g., negative affect, intrusive thoughts, and psychological distress), whereas goal reengagement is related to less negative as well as more positive aspects of well-being [e.g., positive affect and life satisfaction; see meta-analysis by Barlow et al., 2019; review by Wrosch et al. (2013)].

### Goal adjustment and cognitive emotion regulation

As described, developmental regulation theories focus on coping with unattainable goals or a reduction of a discrepancy between an actual- vs. target-situation via cognitive processes. If well-being and self-esteem maintenance are central functions of these processes, this entails the regulation of emotional states. Beyond their constitutive role in well-being, emotions serve as an incentive for goal pursuit and are generated as a result of the discrepancy between target and actual situation, which might channel how to deal with this situation (*cf*. Klinger, 1975; Carver and Scheier, 1990; Kunzmann et al., 2014; Heckhausen et al., 2019; Silvestrini and Gendolla, 2019). Conceptually independent from developmental regulation theories, various models of emotion regulation focus more directly on systematic influences on emotional states (recent overview: McRae and Gross, 2020). Emotion regulation “refers to attempts to influence which emotions one has, when one has them, and how one experiences or expresses these emotions” (Gross, 2015, p. 5; see also Gross, 1998). Accordingly, regulation of the emotion trajectory is the goal itself (Gross and Barrett, 2011), although this again might serve some other goals (Gross, 2015), in particular supporting goal pursuit or disengagement.

Various emotion regulatory processes have been proposed (Gross, 1998, 2015), among them several cognitive emotion regulation strategies. According to Garnefski and Kraaij (2007), “cognitive emotion regulation refers to the conscious, cognitive way of handling the intake of emotionally arousing information” (p. 141) and individuals can use them to regulate their emotions in response to stressful, threatening, or traumatic life events (see, e.g., Andami et al., 2023). Garnefski and Kraaij (2007) identified nine different cognitive coping strategies that individuals may use to regulate emotions in these situations, as assessed by the Cognitive Emotion Regulation Questionnaire (CERQ). Some of these strategies show terminological and conceptual overlap with processes of goal adjustment as proposed in the accommodative coping mode, compensatory secondary control processes as well as goal reengagement but not with goal disengagement in the goal adjustment theory. In particular, *positive reappraisal* refers to attributing positive meaning to a negative event. The assessed severity of an event can be reduced by comparing it to other events (*putting into perspective*). The person can try to come to terms with the situation (*acceptance*) or thoughts can be redirected to more positive issues (*positive refocusing*). In contrast, other cognitive emotion regulation strategies, such as *refocus on planning* (thinking about concrete steps to deal with the event), *self-blame* (blaming oneself for the situation), *other-blame* (blaming other people or the environment for the situation), *rumination* (thinking about the feelings and thoughts associated with the event), or *catastrophizing* (focusing on the distressing aspects of the situation) share less conceptual overlap or point to the opposite of what goal adjustment processes achieve.

So far, the three theories of developmental regulation have not been investigated in one study concerning their association with cognitive emotion regulation as well as their relative associations with regulatory outcome measures, such as various facets of well-being or self-efficacy. Initial evidence that accommodation partly involves aspects of cognitive emotion regulation was provided in a study by Thomsen (2016) in an adolescent sample, in which several CERQ subscales were assessed as well. While accommodative coping showed positive associations with the CERQ subscales *acceptance*, *positive refocusing*, and *putting into perspective*, it correlated most highly with the *positive reappraisal* subscale.

### Present study

Although all three theories share a common conceptual point of departure [i.e., goal adjustment as a functional response to (the experience of) blocked goals or the threatening conditions], and although all three approaches postulate individual differences with respect to the preparedness and inclination to adjust goals, they differ in several assumptions with respect to functions and interrelations of subprocesses of goal adjustment. Moreover, the intersection with, or difference to, processes of cognitive emotion regulation is expected to vary as well. Hence, the present cross-sectional online study had three aims.

First, we tested the relationship between the three goal regulation approaches concerning goal adjustment processes. Based on the conceptual analysis and the empirical findings of Haase et al. (2013), we expected processes of accommodation and compensatory secondary control to be positively associated in the present study as well. Given that accommodation and goal disengagement conceptually focus on the same functional effect, one could assume a positive relationship. However, considering the operationalization of the constructs, while the measurement of accommodative processes in the FGA scale consists of processes facilitating goal disengagement but no items on goal disengagement itself, the GD scale of the GAS focusses only on the ease of goal disengagement (cognitive-affectively and behaviorally). Therefore, only a small positive correlation is to be expected, if at all. In contrast, the subscale CSC also includes items on goal disengagement itself, which is why CSC and GD might show a higher positive correlation. Concerning the relationship between accommodation and goal reengagement, we expected a positive correlation because the FGA scale also includes items on goal reorientation. The same is expected for the CSC and GR. Beyond the basic relationships between the scales, we tested whether a model with a superordinate factor representing the commonality of the scales fits the data better than a model with correlated but independent factors representing the scales.

Second, we examined the relationship of the three approaches with regulatory outcome measures such as well-being and self-efficacy and their relative predictive value over and above the other respective processes. Based on the findings described above, we expected positive correlations of FGA, CSC, and GR with measures of positive affect, self-esteem, life satisfaction, and self-efficacy, and negative associations with negative affect. For GD, we expected a negative correlation with negative affect. An open question is whether the regulatory processes of the three theories cover the same covariance with the measures of well-being or whether each has some unique predictive value over and above the respective other processes. Assuming that the goal adjustment processes postulated in the three models are sufficiently different conceptually and empirically, the empirical question then arises as to their respective partial functionality: To what extent can the individual processes make an independent contribution to the stabilization of the self and well-being over and above the respective other processes? The premise of the conceptual and empirical independence of these processes and their respective assessments is, as alluded to above, gradual rather than categorical. At the same time, this question can also be addressed in a more empirically informed manner through competing predictions (multiple regression). Careful attention, however, will need to be paid to the extent to which common variance explanations arise through empirical overlap (similar item formulations) or conceptual overlap.

Third, we explored the relationship between the three approaches and cognitive emotion regulation strategies and their specific contributions to predict regulatory outcome measures. Based on conceptual considerations as well as partial empirical findings, we assumed that accommodative coping as well as compensatory secondary control processes would correlate positively with the CERQ subscales *acceptance*, *putting into perspective*, and *positive reappraisal*. Moreover, *positive refocusing* was assumed to be positively correlated with accommodative coping, compensatory secondary control processes as well as goal reengagement. Given that goal disengagement is assumed to be facilitated by the processes conceptualized as facets of accommodative coping or compensatory secondary control (Wrosch et al., 2003b), there should also be positive correlations between these CERQ subscales and goal disengagement. How the conceptually unaddressed CERQ strategies relate to processes of goal adjustment cannot be derived from either of these models. Moreover, assuming conceptual and empirical overlap between some of the CERQ subscales and processes of goal adjustment, the respective unique predictive value concerning regulatory outcome measures is an open question and will be explored in a multiple regression.

## Methods

All research reported in this paper was approved by the authors’ University Review Board (no. 195) and was conducted in accordance with the ethical standards of the Declaration of Helsinki. The study was not preregistered. Data, materials, and Supplementary Material can be obtained on OSF: https://osf.io/yrf6m.

### Sample

All participants were recruited via the online platform Prolific, which allowed pre-selection of only English speakers over the age of 18. In order to achieve a large and heterogeneous sample, no further exclusion criteria were specified. A sample size of *N* = 500 completed questionnaires was prespecified.

In total, 528 individuals clicked on the link to the survey. Of these, 501 participants completed the survey. To ensure data quality, we applied measures of data screening (DeSimone et al., 2015). At the end of the survey, two participants indicated that they did not answer seriously and accurately to all statements of this questionnaire. Two additional questions placed in the first third and the second third of the survey asked for selecting a specific response (“for technical reasons”); 433 participants replied correctly to both questions (451 answered correctly to one question, 469 to the other). That is, 86% of the completed surveys were included in the analyses. Included participants did not significantly differ from excluded participants in terms of age, gender, or highest educational level, *p*s > 0.474.

The present sample was 28.9 years old on average (19–67 years, *SD* = 8.4 years). Half of the participants identified as female, 49% as male, and three participants as other or not wanting to answer. The majority of the sample had a high level of education with either A-levels (10.2%) or university degree (52%). Ten percent of the sample reported being unemployed/seeking employment, while the others were either in school/apprenticeship/at university (34%) or otherwise working. Participants were recruited across the world, with most of them living in Europe (top 3 recruitment countries: Portugal: 21%, Poland: 16%, Italy: 12%) but also in South Africa (21%). In 91% of the sample, mother and/or father had migrated from another country.

### Procedure and measures

Participants first confirmed their consent to the handling of their data and their participation in the study. They then provided information on demographic variables. Ensuing they were asked to answer several questionnaires. We describe these questionnaires in the following in their order of appearance. A full list of collected questionnaires is provided on OSF.

#### Processes of developmental regulation

##### Two-process model

We employed the Tenacious Goal Pursuit and Flexible Goal Adjustment Scales (Brandtstädter and Renner, 1990). Fifteen statements were given for each of the Tenacious Goal Pursuit (TGP) and Flexible Goal Adjustment (FGA) subscales. The TGP subscale consists of six directly formulated and nine inversely formulated items, whereas the FGA subscale comprises 11 directly formulated and four inversely formulated items (e.g., “I usually find something positive even when giving up something I cherish”). Participants rated the extent to which they agree with each statement on a 5-point Likert-type scale ranging from 1 (*fully disagree*) to 5 (*fully agree*). Internal consistency was Cronbach’s α_TGP_ = 0.86 and α_FGA_ = 0.81.

##### Motivational theory of life-span development

To capture the constructs of the lifespan theory of control (Heckhausen and Schulz, 1995), the Optimization in Primary and Secondary Control Scales (OPS; Heckhausen et al., 1998) were used. The Optimization (OPT) scale consists of 12 items and the four scales Selective Primary Control (SPC), Selective Secondary Control (SSC), Compensatory Primary Control (CPC), and Compensatory Secondary Control (CSC; e.g., “When I get into a difficult situation, I remind myself that in many ways I am better off than other people.”) each consist of eight items. These were assessed using a five-point Likert-type scale ranging from 1 (*almost never true*) to 5 (*almost always true*). Cronbach’s alphas were *α*_OPT_ = 0.79, *α*_SPC_ = 0.89, *α*_SSC_ = 0.83, *α*_CPC_ = 0.81, and *α*_CSC_ = 0.68.

##### Goal adjustment theory

Goal adjustment capacities were assessed using the 10-item Goal Adjustment Scale (GAS; Wrosch et al., 2003b). Participants were asked to report how they typically react when they are unable to achieve an important goal in their life and must stop pursuing it. Goal disengagement (GD) capacity was assessed with four items, two of which were inversely worded (e.g., “It’s easy for me to reduce my effort toward the goal.”). Goal reengagement (GR) capacity was surveyed with six items (e.g., “I seek other meaningful goals”). Responses were rated on a five-point Likert-type scale ranging from 1 (*almost never true*) to 5 (*almost always true*). Internal consistency was Cronbach’s *α*_GD_ = 0.73 and *α*_GR_ = 0.85.

#### Emotion regulation

##### Cognitive emotion regulation

Specific cognitive emotion regulation strategies were assessed using the Cognitive Emotion Regulation Questionnaire (CERQ; Garnefski and Kraaij, 2007). The CERQ includes 36 items and is composed of the following nine subscales, each consisting of four items: (Self-Blame, Acceptance, Rumination, Positive Refocusing), Refocusing on Planning, Positive Reappraisal, Relating, Catastrophizing, and Blaming Others. The use of these cognitive emotion regulation strategies was rated on a five-point Likert-type scale ranging from 1 (*almost never*) to 5 (*almost always*). Cronbach’s alphas ranged between *α*_AC_ = 0.74 and *α*_PRea_ = 0.93. Internal consistency was beyond 0.70 for all subscales; for six subscales it was >0.80.

#### Regulatory outcome measures

##### General self-efficacy

The Self-Efficacy scale developed by Schwarzer and Jerusalem (1995) was used to measure general perceived self-efficacy. The scale consists of 10 statements (e.g., “I can always manage to solve difficult problems if I try hard enough.”) on a 4-point Likert-type scale ranging from 1 (*not at all true*) to 4 (*exactly true*). In this study, a high Cronbach’s alpha was obtained, with α = 0.90.

##### Self-esteem

Participants’ self-esteem was assessed using the Rosenberg Self-Esteem Scale (RSES; Rosenberg, 1965). This consists of 10 statements dealing with general feelings about oneself and contains five directly (e.g., “I take a positive attitude toward myself.”) and five inversely (e.g., “I feel that I do not have much to be proud of.”) worded items. Although the Rosenberg scale was originally designed as a Guttman scale, it is often used as a Likert scale (e.g., Schmitt and Allik, 2005; Boduszek et al., 2013). In accordance with this approach, a four-point Likert scale ranging from 1 (*strongly disagree*) to 4 (*strongly agree*) was used for the present study, such that higher scores reflect a more positive self-esteem. Cronbach’s alpha was very good, with α = 0.90.

##### Positive and negative affect

To assess respondents’ positive and negative affect, the 20-item Positive and Negative Affect Schedule (PANAS; Watson et al., 1988) was used. The Positive Affect and Negative Affect subscales each consist of 10 words describing various positive (e.g., enthusiastic, interested) and negative (e.g., scared, upset) feelings and emotions, respectively. Participants were asked to rate the extent to which they generally feel this way using a five-point Likert-type scale ranging from 1 (*very slightly or not at all*) to 5 (*extremely*). Accordingly, higher scores represent higher levels of positive or negative affect. Cronbach’s alpha was very good, with *α*_PA_ = 0.90 and *α*_NA_ = 0.90, respectively.

##### Life satisfaction

Life satisfaction was measured using the single item “In general, how satisfied are you with your life?” (Cheung and Lucas, 2014). Participants were asked to rate this on a 4-point Likert-type scale ranging from 1 (*very dissatisfied*) to 4 (*very satisfied*), such that higher scores represent higher life satisfaction.

### Statistical analyses

Data were analyzed using structural equation modeling (SEM) with M*plus* version 8.1.5 (Muthén and Muthén, 1998–2012). We used comparative fit index (CFI: > 0.90) and root-mean-square error of approximation (RMSEA: <0.08) as indicators of reasonable model fit. Prior to parceling, respective items were reverse-coded. For most latent variables, items were then parceled into three indicators. However, goal disengagement had two parcels and compensatory secondary control processes had four parcels, consisting of two items each. Moreover, the latent variable of each CERQ subscale had the four items of each subscales as indicators. Outputs of the measurement and structural models are available as Supplementary Material on OSF as text files.

## Results

An overview of the correlations between the latent variables is provided in Table 2 (for manifest variables, see Supplementary Table S1 for means, standard deviations, and Supplementary Table S2 for correlations in supplementary material on OSF).

**Table 2:** Correlation of latent variables.

		1	2	3	4	5	6	7	8	9	10	11	12	13	14	15	16	17
1	ACCO	–																
2	CSC	**0.43**	–															
3	DIS	0.02	−0.00	–														
4	REE	**0.35**	**0.47**	**0.21**	–													
5	ACC	**0.29**	**0.14**	0.08	**0.28**	–												
6	PosRef	**0.33**	**0.39**	−0.01	**0.24**	**0.21**	–											
7	RefPlan	**0.54**	**0.43**	**−0.25**	**0.41**	**0.34**	**0.43**	–										
8	PosReap	**0.68**	**0.44**	**−0.17**	**0.33**	**0.32**	**0.42**	**0.71**	–									
9	PutPers	**0.36**	**0.49**	−0.11	**0.25**	**0.31**	**0.41**	**0.39**	**0.53**	–								
10	RUM	**−0.26**	0.10	**−0.13**	**0.13**	**0.22**	−0.00	0.11	0.07	0.04	–							
11	CAT	**−0.44**	0.04	−0.10	0.03	**−0.12**	**−0.17**	**−0.17**	**−0.12**	**−0.27**	**0.47**	–						
12	S-blame	**−0.48**	**−0.21**	−0.06	−0.05	0.09	**−0.24**	**−0.22**	**−0.29**	−0.09	**0.47**	**0.39**	–					
13	O-blame	**−0.11**	**0.25**	0.11	0.05	0.00	**0.13**	0.05	−0.05	−0.08	**0.11**	**0.35**	0.04	–				
14	SelfEF	**0.64**	**0.41**	**−0.35**	**0.39**	**0.34**	**0.27**	**0.64**	**0.59**	**0.33**	−0.10	**−0.24**	**−0.29**	−0.03	–			
15	SelfES	**0.68**	**0.38**	**−0.18**	**0.29**	**0.17**	**0.31**	**0.62**	**0.59**	**0.28**	**−0.25**	**−0.41**	**−0.50**	−0.10	**0.70**	–		
16	PosA	**0.60**	**0.43**	**−0.32**	**0.37**	**0.19**	**0.34**	**0.65**	**0.63**	**0.24**	−0.01	**−0.17**	**−0.32**	−0.06	**0.70**	**0.78**	–	
17	NegA	**−0.59**	**−0.17**	0.02	−0.08	−0.12	**−0.17**	**−0.32**	**−0.40**	**−0.26**	**0.49**	**0.47**	**0.53**	**0.18**	**−0.48**	**−0.65**	**−0.36**	–
18	LS^a^	**0.51**	**0.31**	0.08	**0.25**	**0.10**	**0.35**	**0.39**	**0.48**	**0.33**	**−0.13**	**−0.28**	**−0.36**	**−0.03**	**0.53**	**0.75**	**0.62**	**−0.51**

^a^Manifest variable. Bold: *p* < 0.05. Goal adjustment measures: ACCO, accommodative coping; CSC, compensatory secondary control; DIS, goal disengagement; REE, goal reengagement. Cognitive emotion regulation strategies: ACC, acceptance; PosRef, positive refocusing; RefPlan, refocus on planning; PosReap, positive reappraisal; PutPers, putting into perspective; RUM, rumination; CAT, catastrophizing; S-blame, self-blaming; O-blame, other-blaming; SelfEff, self-efficacy; SelfES, self-esteem; PosA, positive affect; NegA, negative affect; LS, life satisfaction.

### Relationship between goal adjustment measures

As can be seen in Table 2 and as expected, accommodative coping and compensatory secondary control were moderately positively correlated. Moreover, there were moderate positive correlations between these scales and goal reengagement. In contrast, we obtained no significant correlation with goal disengagement. Goal disengagement and reengagement were weakly positively correlated.

We conducted a model test including zero-order correlations between the four latent variables. The model showed insufficient model fit [*Χ*^2^(48) = 229.58, *p* < 0.001, RMSEA = 0.093, 90% C.I. [0.082; 0.106], CFI = 0.91]. Comparing it to a model where all latent variables load on one higher factor of *goal adjustment* resulted in slightly worse model fit [*Χ*^2^(50) = 242.28, *p* < 0.001, RMSEA = 0.094, 90% C.I. [0.083; 0.106], CFI = 0.90; Δ*Χ*^2^(2) = 12.7, *p* = 0.002].^2^ Accordingly, while most of the goal adjustment processes shared some variance as indicated by the moderate correlations, they do not seem to be identical or substitutable. In particular, goal disengagement stands out.^3^

### Relationship between goal adjustment and regulatory outcome measures

In a first step, we examined the correlations between the goal adjustment variables and regulatory outcome measures to investigate their basic associations (Table 2). Accommodative coping was strongly positively correlated with self-efficacy, self-esteem, positive affect, and life satisfaction and had a strong negative correlation with negative affect. Compensatory secondary control and goal reengagement demonstrated the same pattern of associations, albeit at a moderate level (and a non-significant correlation between goal reengagement and negative affect). Interestingly, goal disengagement showed small to moderate negative correlations with self-efficacy, self-esteem, and positive affect, that is, higher goal disengagement capacity was related to lower self-efficacy, self-esteem, and positive affect.

In a second step, we wanted to examine the relative contribution of each goal adjustment variable of statistically explained variance. Therefore, we conducted multiple regression analyses for each latent outcome measure as criterion and latent goal adjustment measures as predictors jointly (Table 3). For each outcome measure, accommodative coping was a significant positive predictor and had the highest standardized coefficients. In contrast, compensatory secondary control was mostly not a significant predictor of any of the outcome variables (except for positive affect). Goal disengagement showed significant negative associations with the outcome measures (except for a non-significant relationship with negative affect). Finally, goal reengagement had unique predictive value for self-efficacy and positive affect. Higher goal reengagement was related to greater self-efficacy as well as greater positive affect. However, it also showed a small positive association with negative affect.

**Table 3:** Multiple regression in a structural equation model including all predictors and criteria.

	Self-efficacy			Self-esteem			Positive affect			Negative affect			Life satisfaction^a^		
	*B*	Beta	*p*	*B*	Beta	*p*	*B*	Beta	*p*	*B*	Beta	*p*	*B*	Beta	*p*
ACCO	**0.60**	0.52	<0.001	**0.76**	0.63	<0.001	**0.75**	0.47	<0.001	**−1.17**	−0.65	<0.001	**0.74**	0.45	<0.001
CSC	0.05	0.06	0.275	0.07	0.07	0.209	**0.15**	0.13	0.024	0.07	0.05	0.438	0.09	0.07	0.239
DIS	**−0.33**	−0.41	<0.001	**−0.18**	−0.21	<0.001	**−0.43**	−0.38	<0.001	0.01	0.01	0.928	**−0.24**	−0.21	<0.001
REE	**0.25**	0.26	<0.001	0.08	0.08	0.129	**0.30**	0.23	<0.001	**0.18**	0.12	0.046	0.14	0.10	0.065
*R* ^2^	0.60			0.52			0.55			0.37			0.32		

^a^Manifest variable. Bold: *p* < 0.05. ACCO, accommodative coping; CSC, compensatory secondary control; DIS, goal disengagement; REE, goal reengagement.

In sum, while compensatory secondary control showed significant correlations with all outcome measures, it had no unique predictive value when taking into account other goal adjustment measures. In contrast, particularly goal disengagement and to some extent goal reengagement demonstrated unique predictive value over and above accommodative coping. However, contrary to expectation, goal disengagement showed negative associations with the outcome measures. Overall, explained variance for the outcome measures was quite high ranging from *R*^2^ = 0.32 to *R*^2^ = 0.60.^4^

### Relationship between goal adjustment, cognitive emotion regulation, and regulatory outcome measures

In a first step, we were interested in the basic correlations between goal adjustment processes and cognitive emotion regulation strategies. As displayed in Table 2, accommodative coping showed significant associations with all CERQ subscales ranging from (some) small to (mostly) moderate and strong correlations. The highest correlation was with *positive reappraisal*. Compensatory secondary control showed a similar pattern of associations, but there was no significant relationship with *rumination* and *catastrophizing*. Contrary to accommodative coping, compensatory secondary coping was also positively linked to *blaming others*. Goal disengagement showed only a few significant links with the CERQ subscales. Notably, these were significant negative correlations with *refocus on planning*, *positive reappraisal*, and *rumination*. Goal reengagement was positively correlated with all CERQ subscales except *catastrophizing* and *blaming others* or *oneself*.

A second question concerned the relative contribution of the CERQ scales besides the goal adjustment variables to predict the regulatory outcome measures and how their inclusion might change the pattern of associations. Therefore, we again conducted multiple regression analyses with the latent variables now including the CERQ scales. As displayed in Table 4, although the inclusion of the CERQ subscales significantly increased explained variance for each outcome measure, the pattern of findings for goal adjustment measures remained basically the same. Yet, while the impact of accommodative coping was reduced, for goal disengagement and reengagement it was comparable to the analyses without consideration of the CERQ subscales. Some CERQ subscales had a unique contribution over and above the goal adjustment variables. Particularly, *refocus on planning* was predictive of higher self-efficacy, self-esteem, and positive affect. In contrast, *self-blame* was linked to less self-esteem, positive affect, and life satisfaction, and higher negative affect.

**Table 4:** Multiple regression in a structural equation model including all predictors and criteria.

	Self-efficacy			Self-esteem			Positive affect			Negative affect			Life satisfaction^a^		
	*B*	Beta	*p*	*B*	Beta	*p*	*B*	Beta	*p*	*B*	Beta	*p*	*B*	Beta	*p*
ACCO	**0.38**	0.33	0.000	**0.35**	0.28	0.001	**0.44**	0.27	0.001	**−0.49**	−0.27	0.003	**0.43**	0.26	0.006
CSC	0.07	0.08	0.229	0.07	0.08	0.224	0.08	0.07	0.318	0.03	0.02	0.769	−0.01	−0.01	0.919
DIS	**−0.31**	−0.39	0.000	**−0.14**	−0.17	0.001	**−0.33**	−0.29	0.000	0.02	0.02	0.716	**−0.24**	−0.21	0.000
REE	**0.20**	0.22	0.000	0.09	0.09	0.093	**0.23**	0.18	0.001	0.07	0.05	0.400	**0.20**	0.15	0.013
ACC	**0.10**	0.15	0.003	−0.04	−0.05	0.263	−0.06	−0.07	0.182	−0.04	−0.04	0.490	−0.07	−0.07	0.196
PosRef	−0.03	−0.06	0.238	0.01	0.02	0.717	0.05	0.07	0.147	0.05	0.06	0.221	0.07	0.09	0.085
RefPlan	**0.12**	0.19	0.009	**0.16**	0.24	0.001	**0.15**	0.17	0.023	−0.08	−0.08	0.339	−0.02	−0.02	0.781
PosReap	0.03	0.04	0.679	0.07	0.09	0.271	**0.17**	0.17	0.050	−0.09	−0.08	0.383	0.12	0.12	0.212
PutPers	−0.02	−0.03	0.607	−0.03	−0.05	0.442	−0.02	−0.02	0.728	−0.13	−0.13	0.053	0.06	0.06	0.369
RUM	−0.10	−0.12	0.053	−0.07	−0.08	0.199	0.03	0.02	0.699	**0.40**	0.30	0.000	0.03	0.03	0.693
CAT	−0.06	−0.06	0.350	**−0.19**	−0.16	0.009	0.04	0.03	0.671	0.10	0.06	0.380	−0.20	−0.12	0.069
S-blame	−0.02	−0.03	0.567	**−0.10**	−0.18	0.000	**−0.08**	−0.11	0.038	**0.17**	0.22	0.000	**−0.10**	−0.13	0.024
O-blame	0.02	−0.04	0.419	−0.02	−0.03	0.490	−0.04	−0.05	0.263	0.08	0.08	0.122	0.05	0.05	0.320
*R* ^2^	0.65			0.63			0.60			0.54			0.37		
Δ*R*^2^	0.05 *F* = 3.99, *p* < 0.05			0.11 *F* = 9.49, *p* < 0.05			0.05 *F* = 3.99, *p* < 0.05			0.17 *F* = 11.80, *p* < 0.05			0.05 *F* = 2.53, *p* < 0.05		

^a^Manifest variable. Bold: *p* < 0.05. Goal adjustment measures: ACCO, accommodative coping; CSC, compensatory secondary control; DIS, goal disengagement; REE, goal reengagement. Cognitive emotion regulation strategies: ACC, acceptance; PosRef, positive refocusing; RefPlan, refocus on planning; PosReap, positive reappraisal; PutPers, putting into perspective; RUM, rumination; CAT, catastrophizing; S-blame, self-blaming; O-blame, other-blaming; SelfEff, self-efficacy; SelfES, self-esteem; PosA, positive affect; NegA, negative affect; LS, life satisfaction. Full model: *Χ*^2^(1617): 3179.30, *p* < 0.001. RMSEA: 0.047, 90% C.I. [0.045; 0.050]. CFI: 0.907. *F*_crit_(13, 415) = 1.74.

## Discussion

Goals are central to human development (Freund and Riediger, 2006; Freund et al., 2019). They shape behavior and influence mental and physical health. Adaptive handling of unattainable goals is relevant for maintaining stability of the self across the lifespan, prevents from continuous failure and associated negative feelings and paves the way for the pursuit of achievable goals and continued development (Wrosch et al., 2003b; Brandtstädter, 2006; Heckhausen et al., 2010, 2019). The present article sought to provide a conceptual and empirical overview and comparison of goal adjustment theories in the realm of developmental regulatory research and their relationship with measures of well-being as well as cognitive emotion regulation strategies.

### Relationship between goal adjustment theories

We first examined the relationship between individual differences with respect to four (partly intersecting) measures of goal adjustment derived from the three developmental regulation theories: accommodative coping, compensatory secondary control processes, and goal disengagement and reengagement. We obtained a moderate relationship between accommodation and compensatory secondary control, which replicates the findings of Haase et al. (2013) as well as Wahl et al. (2005). Moreover, both were also positively associated with goal reengagement. These findings point to common processes of coping with unattainable goals and their threatening impact, which Haase et al. (2013) summarized as goal disengagement [albeit without considering Wrosch et al. (2003b) goal adjustment scales]. However, when the relationship of these processes to Wrosch et al.’s (2003b) goal disengagement scale is considered, the question arises to what extent this terminology is appropriate and whether they actually refer to the same process(es). Goal disengagement in the goal adjustment theory (Wrosch et al., 2003a,b) refers to the capacity of abandoning efforts to pursue a blocked goal and dissolving commitment to the goal. While Wrosch et al. (2003b) argue that use of self-protective processes (e.g., downward comparisons) facilitates goal disengagement, we did not obtain positive correlations with accommodative coping or compensatory secondary control processes, which comprise these self-protective processes. This finding rather points to the special status of goal disengagement also found by Loidl and Leipold (2019, 2022), where the subscale goal disengagement of their newly created ACCO-5 scale correlated only weakly or not at all with other processes posited as relevant in the goal adjustment process.

The lack of a correlation might indicate that goal disengagement is not closely related to other processes of goal adjustment. That is, dissolving commitment and cessation of behavioral pursuit might be happening partially independent from other processes of goal adjustment that ensure stability of the self and well-being in the face of unattainable goals. Moreover, if someone can easily let go of an unattainable goal, there might be no need to reappraise the situation or use self-serving comparisons. However, one might wonder what allows this easiness and which costs are associated. Potentially, individuals indicating higher goal disengagement capacity set generally less self-relevant goals which is assumed to make it easier to let go of this unattainable goal (Brandtstädter and Rothermund, 2002). It could also – at least to some extent – reflect a tendency to give up more easily. Given that unattainability oftentimes is not a given fact but a question of how the situation is interpreted, giving up easily might prevent these individuals from experiencing success in goal pursuit, perceiving themselves as self-efficacious, or experiencing positive affect. It might be that the goal disengagement subscale at least partially or in some situations is also sensitive for these aspects of giving up goals without the beneficial aspects of being able to let go of an unattainable goal.

### Relationship between goal adjustment and regulatory outcomes

This interpretation is supported by findings concerning the relationship between the goal adjustment scales and regulatory outcome measures. Accommodative coping, compensatory secondary control, and goal reengagement replicate previous study results demonstrating the beneficial effects of goal adjustment for coping with unattainable goals (Brandtstädter, 2007; Barlow et al., 2019; Heckhausen et al., 2019). It is important to note that the findings presented here on correlations with indicators of personal well-being are cross-sectional and therefore cannot be interpreted as causal straight away. However, this problem is less serious for the aim of the present study because we are not primarily concerned with examining causal effects, but with examining differential correlations between the various subscales and overall scales, both among themselves and with external indicators. Therefore, the question of the direction of the causal relationship is not critical. As expected, we obtained positive relationships with positive affect, life satisfaction, self-esteem, and self-efficacy and a negative association with negative affect. In contrast, higher goal disengagement was linked to less positive affect, self-esteem, and self-efficacy. These findings contradict Barlow et al.’s (2019) overall-effect of a small negative relationship between goal disengagement and negative indicators of well-being as well as a non-significant overall-effect for positive indicators. However, a closer look at their analysis reveals that there is a large heterogeneity of the effects. The authors identified some moderators (being at-risk for depression, age) but additional moderators seem to be at play that might also be relevant for the findings in our study (e.g., a sensitivity of the scale to also reflect learned helplessness or pessimism, which might be measured if other goal adjustment processes are covered within the same study by additional scales). Our study results corroborate the findings of Loidl and Leipold (2019, 2022) who employed separate subscales to measure facets of accommodative coping and found partial negative correlations for their goal disengagement subscale. In their longitudinal study, they also found that an increase in goal disengagement was associated with a decrease in self-efficacy (and decrease in goal importance; Loidl and Leipold, 2022).

Overall, these findings emphasize that ease of goal disengagement capacity *per se* – at least measured in this way – does not seem to be unequivocally related to better well-being in the context of unattainable goals. For example, experienced autonomy in the process of goal disengagement has been demonstrated to be relevant in the progress of actual disengagement and related well-being as well (Holding et al., 2020, 2022). When experiencing autonomous motivation to disengage (i.e., identifying with the decision) in contrast to controlled motivation to disengage (i.e., feeling forced to let go), this is associated with greater progress in disengaging from specific goals, and higher well-being.

Our findings also suggest that other processes of goal adjustment than goal disengagement seem to be more relevant in coping with unattainable goals – reappraisal of the situation, devaluing the goal, revaluation of alternative goals, downward-comparisons. These processes are comprised in the scales that operationalize accommodative coping, compensatory secondary control, and goal reengagement. When considered together in their predictions of positive and negative indicators of well-being, particularly accommodative coping stands out as a significant predictor,^5^ but goal reengagement contributes unique variance for some outcome measures as well. This might be the case because goal reengagement is barely considered in the scale measuring accommodative coping.

### Relationship between goal adjustment and emotion regulation

The aforementioned relevant processes in coping with an unattainable goal share resemblance with some cognitive emotion regulatory strategies (Garnefski and Kraaij, 2007) both on a conceptual and measurement level. Our findings of moderate to strong correlations between accommodative coping and compensatory secondary control (and also goal reengagement) with the ER strategies acceptance, positive refocusing, positive reappraisal, and putting into perspective support this argument empirically. The highest correlation being between accommodation and positive reappraisal replicates the finding of Thomsen (2016), who studied an adolescent sample, in an adult sample. Going beyond Thomsen (2016), we also examined the relationship with other cognitive emotion regulation strategies and found negative associations with rumination, catastrophizing, self- and other-blaming particularly for accommodative coping. It seems that the scale flexible goal adjustment (Brandtstädter and Renner, 1990) measuring accommodative coping comprises several processes also theorized as cognitive emotion regulatory processes. When considered together in a multiple regression, accommodative coping remains a strong predictor of the considered outcome variables, albeit somewhat reduced in its predictive power. For almost all criteria, the strategy refocus on planning is also a significant unique predictor, which may not be covered by the goal adjustment measures. This strategy focuses more on changing the situation and therefore rather belongs to the realm of staying committed to the goal. In addition, the strategy self-blame was significant for four criteria. While the likelihood of, for instance, rumination or catastrophizing, seems to be reduced given high accommodation, blaming the self for not achieving a goal seems to be an independent process associated with less self-esteem, positive affect, and life satisfaction, and higher negative affect.

Overall, the question arises to what extent goal adjustment and cognitive emotion regulation represent concepts that can be clearly distinguished from one another. In both cases, a restructuring of perception or evaluation of the situation takes place. While goal adjustment aims at coping with the discrepancy between the actual and the target state (i.e., dealing with the unattainability of the goal), the idea of emotion regulation is to regulate the handling of emotions. However, both families of regulations seem to make use (or consist) of very similar processes. Moreover, emotions usually arise in the context of unsuccessful goal pursuit. Does it matter for the effectiveness of the processes on which basis they are used, i.e., would it make a difference if someone tried to (explicitly) regulate his or her emotions vs. cope with the unattainability of the goal? How is this situation represented by the individual and which role does awareness play in the application of these processes? Moreover, although we have carved out that some cognitive emotion regulation strategies share resemblance with goal adjustment processes, the role of other emotion regulatory strategies for the goal adjustment process is still unclear. For example, does avoidance prolong the disengagement process or does it facilitate it? It helps at least temporarily not to have to experience the unpleasant feelings. Moreover, distance from actual goal pursuit might enable goal devaluation or refocus on other goals in the same way as shelving does (Mayer and Freund, 2022). However, the situation that is avoided might not be solved and still evoke unpleasant emotions, once the unattainable goal situation cannot be avoided anymore.

### Limitations

The present study is limited by several aspects with regard to the scope of the interpretation of its results. The design of the study is determined by the central research objective of investigating the internal relationships between the empirically assessed facets of goal adjustment and going beyond previous studies in several respects. The cross-sectional correlational design of the present study prevents causal interpretations of the functional role of goal adjustment processes. However, as mentioned above, this is not critical for the purpose of the study because we were interested in the basic associations between the different goal adjustment scales and their unique associations with indicators of well-being and quality of life. Still, to investigate their functional role, experimental studies in particular would be desirable here (Kappes and Schattke, 2022; Rühs et al., 2022).

A point common to all three models and their reviews (including the present study) is the use of self-report data (questionnaires). The data used in these studies (and also here) are therefore also limited by the limitations that self-report data are always subject to. For the processes of goal adjustment addressed here, this concerns less the aspect of possible biases due to social desirability and more the plausible limitation due to the limits of accessibility of adjustment processes through introspection and the resulting limitation of the range and validity of self-reports. The use of supplementary methods, such as implicit cognitive measures (e.g., implicit association test; Rühs et al., 2024), would be important here. However, the complex question of validating such implicit and other indirect indicators is a considerable challenge, both methodologically and theoretically.

The question of the extent to which processes of goal adjustment might actually be influenced by sociocultural factors has not yet been discussed theoretically (in any of the three models), nor (to our knowledge) empirically investigated. It is very plausible to assume that the content of goals pursued by individuals (in the context of their development) is strongly influenced, and certainly often shaped, by sociocultural constellations (Grouzet et al., 2005; Oettingen et al., 2008), but it is less clear whether this also affects the processes of their change. This aspect is also an important challenge for future studies.

### Lessons learned: conceptual consequences

The findings presented here support several conclusions. On the one hand, the heterogeneous findings especially with respect to the functionality of goal disengagement are theoretically informative. If goal adjustment is a complex way of mitigating (perceived) goal blockages and threats to the self (in particular, the consistent predictions of accommodative coping in this and numerous other studies provide very convincing evidence for this), then it is obviously worth analyzing the different facets of this adaptation in more detail (*cf*. Loidl and Leipold, 2019). In particular, the point that certain subaspects (in particular, goal disengagement), if considered separately, may not necessarily be functional, i.e., may not be a sufficient condition for stabilization of self or well-being, requires more theoretical attention than in previous work. At the same time, it is noteworthy that it is neither individual aspects of goal adjustment nor specific configurations of individual aspects that ensure the functionality of goal adjustment, but the variability of possible adaptive alternatives. It suggests that there may be numerous functional equivalents for a successful and functional goal adjustment depending on the situation: for example, downward comparison may have a similar (stabilizing) effect as forms of reframing (which, for example, focus on the gain in the loss without denying the loss). Accordingly, Werner et al. (2022) have provided evidence on the relevance of having access to a strategy repertoire. This could explain that flexible goal adjustment – as the most heterogeneous scale – makes the strongest contribution of the scales considered here in predicting indicators of stability and well-being. This, in turn, again highlights that accommodation is not a singular process, but at least a “family” of processes (Skinner and Zimmer-Gembeck, 2007, 2016; Greve and Kappes, 2023). However, looking again more closely, the common conceptual core of these processes (i.e., goal adjustment) suggests that the members of this family share a response mode in dealing with unattainable goals.

At the same time, however, it is also clear that the study approach focused on here cannot clarify the adaptive processes that are actually effective, nor can it even adequately measure them (the scales do not even reflect their model’s conceptual basis satisfactorily). All scales used here have two shortcomings. First, they all do not capture the actual addressed ability (or readiness) for goal adjustment itself, but only the subjective self-assessment with respect to this ability. Second, even if one conceded this point (self-perception should correlate highly with actual ability, *ceteris paribus*), at best the disposition is captured, not the actual process. Predictions based on disposition, however, can by no means provide (or test) the causal explanation sought, but at best confirm the assumption that the disposition is sufficiently stable across time and situations (Greve and Kappes, 2017). Thus, experimental work is needed that experimentally varies goal setting and goal blocking on the part of IVs, and then first situationally captures changes in preference structures and evaluations, and then tests the functionality of this adaptation for indicators of self and well-being (Mayer and Freund, 2022; Rühs et al., 2022). Such an approach could simultaneously resolve another limitation of the findings presented here: The correlative relationships reported here do not test whether an adaptive response to specific experiences of loss or threat secured the level and stability of the indicators considered. The correlation fit this conjecture (and contrary correlations would have challenged it), but they do not, especially as synchronous correlations, provide a rigorous test of this conjecture.

The reference to the limitation that in the questionnaire format used here (as in the vast majority of previous research on the aforementioned theories of developmental regulation) initially only self-report can be recorded is still resolvable in terms of testing the theoretical prediction that also and especially self-construals (ideal selves) can be adjusted in the course of accommodative adjustments in order to stabilize the self, using longitudinal formats (for an exemplary approach see Marek et al., 2022). More challenging, both theoretically and methodologically, is the question of whether goal adjustment processes might be wholly or partially removed from conscious awareness (and reflection). While Heckhausen et al. (1998, 2019; but see Poulin et al., 2005) have variously emphasized the consciousness and controllability (“secondary control”) of goal adjustment (CSC), the two-process model repeatedly emphasizes that accommodative forms of reaction and regulation typically cannot be intentionally directed, are hardly controlled, and often can only be retrospectively perceived (Brandtstädter and Rothermund, 2002; Brandtstädter, 2006, p. 554). Thus, these processes cannot be conceptualized as intentional (this applies to the talk of “emotion-regulatory strategies” in the same way – they, too, are rarely consciously controllable, thus hardly aptly labeled with “strategy”). Some efforts have been made to complement self-reports in goal adjustment research with implicit measures (e.g., Greve and Wentura, 2003; Rühs et al., 2024). Here lies an important task for future theoretical and empirical work.

## Conclusion

While the importance of goals, the abilities to pursue them, the orientation they provide, for the development of individuals has long been emphasized, the question of how people can deal with goal blockages appropriately and functionally has only recently attracted attention. However, the ability to turn to other goals when a (seemingly) insurmountable blockage occurs is apparently no less functional for successful development. This ability, however, involves a variety of components that presumably achieve their regulatory capacity and functional strength only in certain configurations. It is not enough to abandon a blocked goal if the negative self-assessments associated with it cannot be changed and if no other sufficiently functionally chosen goal can take its place. We do not know which (varieties of) constellations of conditions are sufficient to alleviate experiences of blockage or loss and to maintain the individual’s quality of life and consistency of one’s self. Likewise, we still do not know in detail which processes do operate within the individual’s system of goals and evaluation in order to achieve (sufficient) stability. The study of the processes necessary or conducive to this is not only of great theoretical but also of great practical importance – and perhaps still underestimated up to now.

A large number of findings (which also agree or converge in important points with the results presented here) suggest that processes of goal adjustment can help to alleviate or even completely compensate for burdens caused by experiences of goal blockages and serious threats and losses. Although the findings presented here show that the three theories compared here are not completely congruent with regard to the processes subsumed under goal adjustment, this does not argue against the goal of combining the theories in an integrative manner - and differentiating the constitutive and causally relevant sub-processes more precisely in the process. If the functionality of goal adjustment processes can be assumed, then it would make sense to pursue three research perspectives with regard to possible application options. First, it would be important to better understand the question of the developmental conditions of individual willingness and ability of goal adjustment: What are the precursor abilities and when can the first indications of this capacity be shown in childhood and adolescence (Lessing et al., 2019; Greve and Kappes, 2023)? There is currently a lack of longitudinal studies in particular that combine different stages of life (from childhood to adolescence and adulthood). Their planning would be the second research perspective that would be relevant for application options. Thirdly, it should then be investigated in particular whether specific funding opportunities can be identified. For example, there is evidence that heterogeneous developmental conditions can be prognostically favorable for a more pronounced ability and willingness to make accommodative goal adjustments (Greve and Thomsen, 2013; Thomsen and Greve, 2013; Greve et al., 2021; Koch et al., 2024). The promotion of accommodative skills and readiness in childhood has hardly been tested to date (Greve et al., 2009); this would represent an important research desideratum.

## Data availability statement

The datasets presented in this study can be found in online repositories. This data can be found here: https://osf.io/yrf6m.

## Ethics statement

The studies involving humans were approved by University of Hildesheim Faculty 1: Educational and Social Sciences’ Review Board. The studies were conducted in accordance with the local legislation and institutional requirements. The participants provided their written informed consent to participate in this study.

## Author contributions

CK: Writing – review & editing, Writing – original draft, Methodology, Formal analysis, Conceptualization. WG: Writing – review & editing, Writing – original draft, Methodology, Conceptualization.

## Footnotes

^1^In the second study, the model of selection, optimization, and compensation (Baltes and Baltes, 1990; Freund and Baltes, 2002) was also included. Since this model does not focus directly on processes of goal adjustment, we do not consider it in this article.

^2^We also conducted a conceptual replication analysis of Study 1 of Haase et al. (2013) where all scales of the dual-process model of accommodation and assimilation (Brandtstädter and Rothermund, 2002) and of the motivational theory of life-span development (Heckhausen et al., 2010) were included. We tested the integrative model of developmental regulation. We replicated the structural model demonstrating that accommodative coping and compensatory secondary control load on one factor, albeit the higher order factor goal disengagement reflected accommodative coping to a lesser extent compared to Haase et al.’s (2013) findings (see Supplementary Table S3 in supplementary material on OSF). A model extended to include Wrosch et al.’s (2003a,b) goal disengagement and goal reengagement as additional latent variables loading on the goal disengagement factor did result in a not positive definite latent variable covariance matrix (psi).

^3^This finding is also mirrored in the factor structure of an exploratory factor analysis when including all items of the four scales and extracting factors based on an e*igenvalue* of ≥1. The items mainly load on a factor representing their respective scale or are distributed across several factors representing parts of the respective scale (see Supplementary Table S4 in supplementary material on OSF). However, the two items of the compensatory secondary control scale concerning goal disengagement load on the factor representing the GD scale.

^4^We also tested and conceptually replicated Haase et al.’s (2013) findings in terms of testing the integrative model’s relationship with measures of well-being (see Supplementary Figure S1 in supplementary material on OSF).

^5^One reason why FGA, the scale measuring accommodative coping, showed the highest correlations and outperformed the other scales in the multiple regressions, might have to do with its somewhat broader scope: The FGA scale comprises 15 items, whereas the CSC consists of only 8, the GD scale has 4 items, and the GR has 6 items. Hence, the FGA possibly covers a broader range of relevant processes. While the scales measuring tenacious goal pursuit (TGP) and flexible goal adjustment (FGA) have been criticized (Mueller and Kim, 2004; Henselmans et al., 2011) because they do not sufficiently distinguish between both response modes and the keying of items plays an important role in response variance, this criticism mostly concerns the TGP. In the present study, the scales FGA, CSC, as well as GR showed good internal consistency. In contrast, GD had only satisfying internal consistency (which, however, would not explain the hypotheses-inconsistent findings for GD).
